# Cerebrovascular accidents association between serum trace elements and toxic metals level, a case-control study

**DOI:** 10.1371/journal.pone.0317731

**Published:** 2025-02-03

**Authors:** Khosro Jamebozorgi, Alireza Kooshki, Mahbobeh Saljoughi, Mohamadjavad Sanjari, Zahra Ahmadi, Seyed Mohammad Mosavi Mirzaei

**Affiliations:** 1 Faculty of Medicine, Zabol University of Medical Sciences, Zabol, Iran; 2 Medical Toxicology and Drug Abuse Research Center (MTDRC), Birjand University of Medical Sciences, Iran; 3 Neurology Department, Vali-Asr Hospital, Birjand, Iran; Tehran University of Medical Sciences, IRAN, ISLAMIC REPUBLIC OF

## Abstract

**Background:**

Cerebrovascular accidents (CVAs) are among the most common complications of patients today. As the prevalence of ischemic CVAs rises, detecting related risk factors is crucial. Metal concentration has previously been considered a major risk factor in several neural complications, and in this study, we will investigate this.

**Methods:**

In this case-control study, 70 CVA (clinically approved ischemic stroke cases by imaging and NIH Stroke Scale (NIHSS)) and 70 individuals with no history of CVA controls were enrolled as the control group. The serum level of several metals, including Fe (Iron), Co (Cobalt), Ni (Nickel), Cu (copper), Zn (Zinc), Mn (Manganese), Pb (lead), Hg (Mercury), has been assessed using Inductively coupled plasma mass spectrometry (ICP-MS) method. Logistic regression (LR) has also been used to determine the association between metals’ levels and CVA occurrence.

**Results:**

As the mean age of the CVA group was 48.68  ±  15.25 years and for the non-CVA group was 47.89 ± 9.65 years, the result indicated that the serum level of Cu and Pb has been statically higher in the CVA group (respectively; P  <  0.001 and P  =  0.002) and Ni level was significantly lower (P  =  0.003). Other measured metals’ levels (Fe, Co, Ni, Mn, Hg) were not significantly different between CVA and non-CVA groups. In the LR model, all Cu, Pb, and Zn metals had a P value of 0.03 and an odd ratio (OR) and confidence interval (CI) of 1.34 (1.02–1.75), 1.19 (1.01–1.39) and 1.01 (1.001–1.02) respectively.

**Conclusion:**

Given that some metals are associated with a higher risk of CVA, researchers and physicians must better understand the risk factors and causes of the burden of CVA. However, further studies with a larger population and investigation of the exact pathogenesis of these metals are needed.

## 1. Introduction

Known as the most common cause of disability worldwide and third in line responsible for deaths globally, cerebrovascular accidents (CVAs) are one of the most common and also fatal diseases that can occur in one person’s lifetime [1–3]. According to the World’s Stroke Organization, there are about 13.7 million new cases of stroke each year, and the prevalence of stroke can vary from 2 to 15% depending on the age [4,5]. Attributed yearly deaths due to CVA were around 5.5 million cases in 2016 and increased to 6.55 million cases by 2019 [6,7]. It is estimated that CVA is the second cause of death and the third cause of death and disability worldwide [8]. In 2019, the prevalence, mortality, and disability-adjusted life-years (DALY) of CVA was estimated to be around 143 million among 12.2 cases of CVA in just one year, which is a significant number [6].

As the estimates for CVAs are expected to go higher, there are well-known risk factors such as dyslipidemia, high blood pressure, smoking, alcohol, lack of stuffiest physical activity, obesity, and unhealthy diet that can majorly alter the chance of a CVA [9]. Some new risk factors, such as environmental and pollutants, affect the prevalence of CVA. For instance, the oxidative stress pathways and production of free radicals are key and most discussed topics, increasing the risk of several diseases, including CVA. Toxic metals such as mercury (Hg) and lead (Pb) are known to alter oxidative stress pathways and increase the level of reactive oxygen species (ROS) [10]. These metals can also alter the risk of CVA indirectly by increasing the risk of diseases related to CVA, such as hypertension, myocardial infarction, cardiac arrhythmia, and thrombosis [11,12].

Regarding the effect of trace elements on the prevalence of CVAs, the data is limited, and their effect and mechanism of probable action still need to be completely understood. A study indicates a higher level of Iron (Fe) and lower levels of serum zinc (Zn), copper [13], and manganese [14] in acute hemorrhagic stroke patients compared to controls [15]. On the other hand, another study reported lower levels of Zn and Mn but lower levels of Cu in stroke patients [16]. Other studies resulted in lower levels of Fe and a higher level of Zn, Pb, and nickel (Ni) serum levels in stroke patients compared to controls [17].

The role of the disbalance of metals in the pathogenesis of stroke is confirmed by the neuroprotective properties of metal-chelation therapy in CVA patients [18].

Therefore, due to the increase in the probability of exposure to toxic and other metals in today’s industrial world and the increase in CVA among all age groups, especially the elderly, in this case-control study, we tend to measure the serum levels of several toxic metals and trace elements in the ischemic CVA patients and compare the results with non-CVA individuals who do not have a history of CVA.

## 2. Method

### 2.1. Study population

This study was a single-center, case-control study conducted in Birjand, Iran, From March 2022 to March 2023 in Vali Asr Hospital, Birjand, Iran.

In this study, 35 CVA (clinically approved ischemic stroke cases by imaging and NIH Stroke Scale (NIHSS)) and 35 individuals with no history of CVA controls were enrolled as the control group between March 2022 and March 2023. The subjects of the case group were selected from among the hospitalized patients with CVA in Vali Asr Hospital, Birjand. The subjects of the control group were selected from people who had no history of stroke, heart attack, high blood pressure, and diabetes. The two groups were matched for age, gender, and occupation. Once the study objectives and confidentiality of the obtained data were explained to the participants, they signed written consent forms for participation. Demographic data, including age, gender, education, marital status, occupation, opium use, and history of smoking were collected from the subjects.

All experiments involving human participants in this study followed the relevant guidelines and regulations. Before the commencement of the study, approval for the experimental protocols was obtained from the Birjand University of Medical Sciences (IR.BUMS.REC.1399.072). Informed consent was obtained from all subjects or their legal guardians before they participated in the study.

### 2.2. Serum collection and analysis

In this study, on May 15th, 2023, patients and controls were referred for blood collection. Case and control individuals consent for participating and donating blood samples for this study. No fasting was required for this test. An individual’s blood was taken by venipuncture of an antecubital vein. Approximetly, 20 milliliters of blood were taken from each patient in a vacuum tube. The individuals were in siting position and according to the protocol. The samples were centrifuged in room temperature within the first 30 minutes, and serum was collected.

Serum samples underwent digestion using a nitric acid and perchloric acid mixture in a 2:1 v/v ratio. Five ccs of each serum sample were transferred into 25-ml glass test tubes for acid digestion. Subsequently, two ccs of 65% pure nitric acid (Merck, Germany) were added to each serum sample, and the mixture was left at room temperature overnight for gradual digestion. Following this, one cc of 72% perchloric acid (Merck, Germany) was introduced to the mixed specimens, and the resulting mixture was subjected to a 4-hour digestion process in a hot water bath (Bain-Marie) at 98 °C until complete digestion was achieved (Dos Santosa et al., 2018). After digestion, the samples were allowed to cool to ambient temperature, and subsequent dilution was carried out using 25 ml of deionized water. Finally, the samples prepared for toxic metal analysis were measured using an Agilent 7900 ICP-MS.

All standard solutions utilized for metal analysis in this study were prepared from Merck standards with a concentration of 1000 ppm. The concentrations of toxic metals (Pb, Cu, Ni, Cr, Co, Fe, Hg, Mn, and Zn) in this investigation were expressed in micrograms per deciliter. The ICP-MS performance parameters were configured as follows: radiofrequency power—1.5 kW; plasma gas flow rate—15 l per minute; carrier gas flow—1.01 l per minute; constituent gas—0.15 l per minute; sample absorption rate—1.7; sample depth—10 mm; detector mode—auto; scan type—peak hopping (three sweeps per reading and three readings per repetition); and scan number—3.

### 2.3. Statistical analysis

Data analysis was conducted using Stata version 17. characteristics of the study population were depicted using means (standard deviation: SD) and numbers (%) for categorical variables. Medians (interquartile range: IQR) were reported for covariates exhibiting a skewed distribution. A comparison of baseline characteristics between individuals with and without ischemic CVA employed the Student’s t-test for continuous variables, the chi-square test for categorical variables, and the Mann-Whitney test for skewed variables. We used logistic regression models to investigate the association between heavy metal levels and the occurrence of CVA. The significance level was set at 5%. Odds Ratio (OR) and Confidence Interval (CI) were used to report the data. For logistic regression, data analysis was conducted using R4.3.

## 3. Results

### 3.1. Characteristics of the study population

The results of the demographic information 70 participants (62/8 Male/Female) with a mean age of 48.25  ±  12.45 years are reported in Table 1, which indicates that the two groups exhibit similarities in age distribution, opium use, and being smokers. No significant difference between the ischemic CVA and non-CVA groups has been observed in any measured demographic data factors.

**Table 1:** Comparison of demographic information between CVA and non-CVA groups.

Variable	CVA group (n = 35)	Non-CVA group (n = 35)	P-value
Age	48.68 ± 15.25	47.89 ± 9.65	0.11
Gender			
Male	30 (85.7)	32 (91.4)	0.70
Material status			
Married	28 (80.0)	20 (57.1)	0.07
Occupation			
Employment	4 (11.4)	5 (14.3)	0.98
Free	6 (17.1)	6 (17.1)	
Retired	5 (14.3)	4 (11.4)	
Driver	10 (28.6)	8 (22.9)	
House-wife	3 (8.6)	4 (11.4)	
Worker	7 (20.0)	8 (22.9)	
opium			
Yes	24 (68.6)	20 (57.1)	0.46
Smoker			
Yes	29 (82.8)	26 (74.3)	0.56

### 3.2. Toxic metals levels comparison between CVA and non-CVA group

The comparison data illustrating the levels of toxic metals in two distinct groups is presented in Table 2. Comparing the fundamental characteristics of the two groups (CVA and non-CVA controls), we observed that individuals with CVA exhibited higher levels of Cu) Z  =  7.17, p  <  0.001 (and Pb) Z  =  3.13, P  =  0.002 (and lower levels of Ni (Z  =  2.95, p  =  0.003) compared to those without history of CVA. Other metals were not statistically different between CVA and non-CVA groups (Fe; Z  =  0.64, P  =  0.52, Co; T  =  0.71, P  =  0.48, Zn; Z  =  1.94, P  =  0.05, Mn; Z  =  0.06, P  =  0.94, Hg; Z  =  1.92, P  =  0.05).

**Table 2:** Comparison of toxic metal levels between CVA and non-CVA groups.

Variable	CVA group (n = 35)	Non-CVA group (n = 35)	Test results*
Fe	370.44 [208.44–479.75]	279.0 [134.0–601.0]	Z = 0.64,P = 0.52
Co	3.03 ± 1.88	2.68 ± 2.19	T = 0.71,P = 0.48
Ni	17.57 [8.00–65.40]	37.00 [20.00–129.00]	**Z = 2.95,P = 0.003**
Cu	117.03 [96.07–196.90]	34.00 [27.00–42.00]	**Z = 7.17,P < 0.001**
Zn	81.61 [67.14–117.45]	73.00 [25.00–106.00]	Z = 1.94,P = 0.05
Mn	9.75 [2.71–16.79]	8.00 [5.00–16.00]	Z = 0.06,P = 0.94
Pb	8.60 [5.50–10.00]	5.15 [3.60–7.06]	**Z = 3.13,P = 0.002**
Hg	0.70 [0.40–1.00]	0.89 [0.64–1.86]	Z = 1.92,P = 0.05

Fe, Iron; Co, Cobalt; Ni, Nickel; Cu, copper; Zn, Zinc; Mn, Manganese; pb, lead; Hg, Mercury.

*: Independent sample t-test and Mann-Whitney U Test were used for intergroup comparison of normality for continuous and skewness for continuous, respectively.

### 3.3. The association between metals serum concentration measurements and CVA using logistic regression (LR) model

The findings from the logistic regression model are outlined in Table 3. The Cu, Zn, and pb variables demonstrated a significant relationship with the likelihood of CVA. An increase of one unit in pb level was associated with a 19% increase in chance of CVA (OR:1.19, 95% CI:1.01–1.39). An increase of one unit in Cu levels was associated with a 34% increase in the chance of stroke (OR:1.34, 95% CI: 1.02–1.75), while a similar increase in Zn levels showed a 1% rise (OR:1.01, 95% CI:1.001–1.02)

**Table 3:** Shows the association between toxic metal levels and the occurrence of CVA as determined by the logistic regression model.

Variable	OR (95%CI)	P-value
Fe	1.001 (1.00–1.002)	0.16
Co	1.09 (0.86–1.38)	0.48
Ni	0.99 (0.99–1.003)	0.20
Cu	1.34 (1.02–1.75)	**0.03**
Zn	1.01 (1.001–1.02)	**0.03**
Mn	1.02 (0.99–1.05)	0.21
Pb	1.19 (1.01–1.39)	**0.03**
Hg	0.96 (0.71–1.30)	0.79

OR: odds ratio; CI: confidence interval;

Fe, Iron; Co, Cobalt; Ni, Nickel; Cu, copper; Zn, Zinc; Mn, Manganese; pb, lead; Hg, Mercury.

The logistic regression model results, categorized by quartiles of toxic metals, are presented in Table 4. People with pb level in the highest quartile had a 9.17 times higher chance of CVA than those in the lowest quartile (OR: 9.17,95% CI: 1.87–44.99). The chance of CVA decreased by 97%, 92%, and 98% in the first, second, and third quartiles of Ni levels, respectively, compared to the lowest quartile (OR:1.85, 95% CI:0.48–7.18, OR:1.48, 95% CI:0.37–5.94, OR:2.61, 95% CI:0.65–10.47). In the upper quartiles, Mn and Hg levels demonstrated a 90% and 86% reduction in the chance of stroke, respectively, compared to the lower quartile (OR:0.04, 95% CI:0.004–0.38, OR:0.24, 95% CI:0.06–1.04).

**Table 4:** Presents the results of the logistic regression model based on the quartiles of toxic metals.

Variable	OR (95% CI)	p-value
Fe		
Quartile 1	2.88 (0.72–11.38)	0.13
Quartile 2	3.36 (0.82–13.72)	0.09
Quartile 3	1.17 (0.29–4.61)	0.83
Co		
Quartile 1	1.85 (0.48–7.18)	0.38
Quartile 2	1.48 (0.37–5.94)	0.58
Quartile 3	2.61 (0.65–10.47)	0.17
Ni		
Quartile 1	0.03 (0.003–0.29)	0.002
Quartile 2	0.07 (0.007–0.65)	0.02
Quartile 3	0.02 (0.002–0.18)	0.001
Zn		
Quartile 1	26.25 (4.13–166.45)	0.001
Quartile 2	8.43 (1.45–48.85)	0.02
Quartile 3	9.37 (1.63–53.62)	0.01
Mn		
Quartile 1	0.04 (0.004-0.38)	0.005
Quartile 2	0.11 (0.01–1.08)	0.06
Quartile 3	0.1 (0.01–0.95)	0.04
Pb		
Quartile 1	0.61 (0.13–2.75)	0.52
Quartile 2	2.04 (0.53–7.79)	0.29
Quartile 3	9.17 (1.87–44.92)	0.006
Hg		
Quartile 1	0.24 (0.06–1.04)	0.06
Quartile 2	0.5 (0.11–2.31)	0.37
Quartile 3	0.14 (0.03–0.63)	0.01

OR: odds ratio; CI: confidence interval;

Fe, Iron; Co, Cobalt; Ni, Nickel; Cu, copper; Zn, Zinc; Mn, Manganese; pb, lead; Hg, Mercury.

## 4. Discussion

The levels of toxic metals have been observed in two groups, indicating that Cu, Zn, and Pb are higher in individuals suffering from CVA than in normal controls. Additionally, our findings indicate that the decrease in Ni levels is associated with an increased risk of ischemic stroke. Individuals with higher lead levels are more likely to have a stroke compared to those in the lower quartile. On the other hand, manganese and mercury levels showed a 90% and 86% reduction in stroke probability compared to the lower quartile. These findings are unique and have not been broadly discussed in similar studies.

Trace elements and toxic metals play a vital role in various physiological processes; however, excessive exposure to toxic metals and toxicity can be catastrophic for human health [19–22]. Evidence suggests that exposure to chemicals, especially toxic metals, also plays a significant role in causing strokes [3,23,24]. Some studies have indicated the potential role of vascular inflammation due to toxic metals and explained that excessive ROS production may activate inflammatory pathways and thus accelerate cerebral small vessel disease progression. Toxic metals pose a serious health risk to humans due to their ability to alter and damage DNA and membranes and disrupt the functioning of proteins and enzymes, which can lead to CVA [25,26].

Other results of this study indicated that increased copper levels were associated with the risk of CVA. Copper is a rare and essential element, yet it is toxic. Copper is exposed to humans through air and soil food, but most of its exposure is due to agricultural matters [27] and meat consumption [28]. Several functions for this trace element have been suggested, such as angiogenesis, neurohormone homeostasis, brain development, and even regulation of gene expression [29]. Though, as mentioned, this element is crucial for health, excessive amounts of Cu can be toxic, like we see in Wilson’s disease, which is a genetic mutation in ATP7B leading to excessive amounts of Cu in the body and, therefore, several neurological problems [30]. A cohort study involving 4035 middle-aged French participants showed that serum copper is associated with a 30% increase in cardiovascular disease (CVD) mortality compared to extreme quartiles (mean concentration of 952.5 micrograms per liter) [31].

Furthermore, the results of several other studies also indicated an association between serum copper levels and increased risk of CVA mortality [32,33]. In this regard, Hu and colleagues also reached a similar conclusion in their study. In this study, which involved 1255 participants matched for age, gender, and location, it was found that baseline plasma copper is positively associated with the risk of ischemic stroke [34]. However, this study was conducted based on plasma copper. Overall, several molecular mechanisms that may be involved in toxic metal-induced stroke have been described, such as oxidative stress, apoptosis, inflammation, cerebral microvascular damage, endothelial dysfunction, matrix metalloproteinase expression, and amyloid angiopathy [23]. Patwa and colleagues also demonstrated in their study that copper leads to amyloid deposition in the brain, which is the primary cause of cerebral amyloid angiopathy [35]. As a 2023 review indicates, a higher level of copper ion in aging populations shows a significant relation between copper and vascular aging [36]. Furthermore, copper also increases blood pressure through oxidative stress and inflammation, and serum copper levels above 130 micrograms per deciliter increase blood pressure sensitivity by 1.99 times. Since many factors influence stroke, further research is needed for a better understanding of the relationship between serum copper and stroke risk.

Another study result is the association between serum zinc levels and stroke. It was found in this study that the higher the level of zinc, the higher the risk of stroke. Some studies have shown that the type of stroke is associated with zinc levels [37]. The results of the Huang study are consistent with the present study’s findings [38]. The study by Huang and colleagues, a meta-analysis study, showed that serum zinc levels in patients with ischemic stroke were significantly higher than those in the control group. However, a significant association between zinc levels and the risk of hemorrhagic stroke was not found [38]. It has been identified that elevated Zinc levels inhibit the absorption of Copper and Iron, increase ROS production in mitochondria, disrupt metabolic enzyme activity, and activate apoptotic proteins. Zinc homeostasis disturbance leads to Alzheimer’s disease, brain damage, ischemic stroke, and other conditions [39].

Interestingly, it is worth mentioning that low zinc levels are associated with stroke. Two other cross-sectional studies also showed that serum zinc levels are significantly lower among stroke patients compared to the control group, and low serum zinc levels are associated with increased severity of acute stroke in stroke patients [13,40]. However, these findings suggest the need for further research in this area and investigation into appropriate dosage.

The results of this study indicate that an increase in lead levels is associated with an increased risk of stroke, and there is a direct correlation between them. Additionally, individuals with higher lead levels had a 9.17 times higher probability of ischemic CVA than those in the lower quartile. A meta-analysis conducted by Bao and colleagues on 38 studies involving 642,014 participants showed that lead is significantly associated with the risk of stroke. However, two other metals (Arsenic and Mercury) had less impact on the risk of stroke. This study also suggested that the association is likely dose-dependent [41].

Furthermore, a case-control study examined the association of 45 mineral elements with stroke in 92 patients. Blood lead levels were significantly higher and were associated with stroke [42]. The studies mentioned above confirm these results. However, one study reported an association between serum mercury and Myocardial Infarction. At the same time, non-significant differences were observed in serum and urine levels of lead, arsenic, and cadmium [43].

What is clear is that lead possesses specific toxic properties in fibrinolysis, proliferation, and extracellular matrix formation in smooth muscle cells and endothelial cells, which can lead to vascular disorders, including arterial sclerosis, in laboratory animals. Furthermore, some animal studies indicate that lead may cause significant damage to cerebral vascular endothelium and disrupt cerebral microvessels’ function by altering cerebral blood flow. Therefore, lead may play a role in developing cerebral atherosclerosis and CVA [44].

The results of this study specified that lower levels of nickel are associated with an increased risk of stroke. In this regard, Li and colleagues [45] and Skalny [14] stated in their studies that nickel and some other metals (such as cadmium, arsenic, mercury, and aluminum) are associated with the pathophysiology of stroke.

The analysis of the results of this study showed that both excessive increase and severe decrease in manganese are associated with ischemic stroke. The study’s results by Alikunju and colleagues [46] are consistent with the present study. Alikunju and colleagues [46] stated in their case report study that rarely, manganese neurotoxicity can present as stroke; only one case in a published report has been documented. This study mentioned that higher levels of manganese in red blood cells are associated with greater accumulation in the brain. In addition to the above cases, Wen and colleagues [47] conducted a case-control study on 1277 individuals to investigate the relationship between several plasma metals and the risk of ischemic stroke. The results of this study are consistent with the present study, indicating that the concentration of various metals, including manganese, in patients with ischemic stroke was significantly higher than in control individuals [47]. Mechanisms of manganese-induced issues have been studied, including oxidative stress, mitochondrial dysfunction, glutamate toxicity, protein misfolding, inflammation, autophagy, mitophagy, endoplasmic reticulum stress, and apoptosis [47–49]. Additionally, another study mentioned that manganese induces proteolytic cleavage of protein kinase C-delta (PKC-δ), one of the essential factors in brain damage, and is also a major factor in the neuroprotective activity of the protein alpha-synuclein [39].

As a strength of this study, this is the first study that assesses these metals in the Persian population. Assessing all these metals together with some of the most common confounding factors like smoking and opium use is the other strength of the study. Some limitations in this study should be taken into account. Firstly, as this is one of the few studies assessing these metals’ levels in CVA patients, the number of cases and ethnicity are limited. Future studies can assess these metals in more cases with various ethnicities to better demonstrate this effect. Secondly, as hemorrhagic stroke is less frequent, the cases were not adequate to form a separate group. Lastly, since our study was a case-control study, determining the cause and effect needs to be more studied in this field. It is suggested that future studies use hemorrhagic stroke patients’ groups to evaluate the difference between hemorrhagic and ischemic stroke patients.

## 5. Conclusion

In conclusion, given that the general population is widely exposed to several metals, even a slight increase in the risk of stroke due to exposure to metals (especially copper) can have significant public health implications. Reducing exposure to multiple metals may help alleviate the risk of stroke.

## Supporting information

S1 Dataset Raw data used for the analysis. (XLSX)
